# Crizotinib-Resistant Mutants of EML4-ALK Identified Through an Accelerated Mutagenesis Screen

**DOI:** 10.1111/j.1747-0285.2011.01239.x

**Published:** 2011-12

**Authors:** Sen Zhang, Frank Wang, Jeffrey Keats, Xiaotian Zhu, Yaoyu Ning, Scott D Wardwell, Lauren Moran, Qurish K Mohemmad, Rana Anjum, Yihan Wang, Narayana I Narasimhan, David Dalgarno, William C Shakespeare, Juan J Miret, Tim Clackson, Victor M Rivera

**Affiliations:** ARIAD Pharmaceuticals, Inc. CambridgeMA 02139, USA

**Keywords:** crizotinib, EML4-ALK, ENU mutagenesis, gatekeeper, resistance

## Abstract

Activating gene rearrangements of anaplastic lymphoma kinase (ALK) have been identified as driver mutations in non-small-cell lung cancer, inflammatory myofibroblastic tumors, and other cancers. Crizotinib, a dual MET/ALK inhibitor, has demonstrated promising clinical activity in patients with non-small-cell lung cancer and inflammatory myofibroblastic tumors harboring ALK translocations. Inhibitors of driver kinases often elicit kinase domain mutations that confer resistance, and such mutations have been successfully predicted using *in vitro* mutagenesis screens. Here, this approach was used to discover an extensive set of ALK mutations that can confer resistance to crizotinib. Mutations at 16 residues were identified, structurally clustered into five regions around the kinase active site, which conferred varying degrees of resistance. The screen successfully predicted the L1196M, C1156Y, and F1174L mutations, recently identified in crizotinib-resistant patients. In separate studies, we demonstrated that crizotinib has relatively modest potency in ALK-positive non-small-cell lung cancer cell lines. A more potent ALK inhibitor, TAE684, maintained substantial activity against mutations that conferred resistance to crizotinib. Our study identifies multiple novel mutations in ALK that may confer clinical resistance to crizotinib, suggests that crizotinib's narrow selectivity window may underlie its susceptibility to such resistance and demonstrates that a more potent ALK inhibitor may be effective at overcoming resistance.

Chromosomal translocations of anaplastic lymphoma kinase (ALK), originally identified in anaplastic large cell lymphoma (1), have now been found in multiple tumor types, including inflammatory myofibroblastic tumors (IMT), and in 3–7% of non-small-cell lung cancers (NSCLC) (2,3). Activating mutations and ALK gene amplifications have also been detected in neuroblastomas (4). Preclinical studies demonstrate that ALK inhibition induces apoptosis and tumor regression in models of ALK-expressing tumors, identifying ALK as a driver mutation and underscoring its potential as a therapeutic target (5). Recently reported data from a phase 1 trial of crizotinib (PF-02341066), a dual MET/ALK inhibitor (6) in ALK-positive patients with NSCLC, revealed significant clinical efficacy (7). Combined with a response in a patient with ALK-positive IMT (8), these data provide clinical validation of ALK as a target and proof-of-concept for the targeted use of ALK inhibitors in ALK-driven tumors.

Treatment for tumors expressing driver kinases with targeted inhibitors commonly leads to acquired resistance because of point mutations in the kinase domain. *In vitro* accelerated mutagenesis screens are powerful methods for identifying such mutations (9,10) and have successfully predicted and recapitulated the spectra of mutations observed clinically – for example, following the treatment for chronic myeloid leukemia (CML) with different BCR-ABL inhibitors (10). In this study, we conducted a mutagenesis screen to identify the potential resistance mechanisms to crizotinib in ALK-driven tumors and determined whether a more potent ALK inhibitor, TAE684 (11), could overcome resistance.

## Methods and Materials

### Cell lines and reagents

H2228, H838, and H23 NSCLC lines were obtained from the American Type Culture Collection (ATCC) and Ba/F3 cells from Deutsche Sammlung von Mikroorganismen und Zellkulturen (DSMZ). ATCC cell lines were authenticated by ATCC's routine Cell Biology Program and were used within 6 months of receipt. Ba/F3 cells were used within 6 months of receipt from DSMZ that authenticates human cell lines by routine multiparameter methods prior to accession. H3122 cells were obtained from NCI with no further authentication made. Crizotinib and TAE684 (Figure S1) were synthesized at ARIAD Pharmaceuticals. Unambiguous structural assignments were made by routine spectroscopic methods including NMR, LC-MS, and CHN analyses.

### *In vitro* cell growth, viability, and signaling

Cells were treated with crizotinib, TAE684, or vehicle (DMSO) for 72 h. The effect on NSCLC growth was assessed using CyQuant (Invitrogen). The concentration causing 50% growth inhibition (GI50) was determined by subtracting the cell count at time zero and plotting relative to vehicle-treated cells. The effect on Ba/F3 cell viability (IC_50_) was assessed using CellTiter-96 AQueous One (Promega, Madison, WI, USA) and plotting viable cells relative to vehicle-treated cells.

Cell lysates prepared after 2-h treatment with compound were analyzed by immunoblotting, using antibodies against p-ALK^Y1604^, total-ALK, p-STAT3^Y705^, p-AKT^S473^, p-ERK1/2^T202/Y204^, p-S6P^T240/244^, or by PathScan Sandwich ELISA against p-ALK^Y1604^ and total-ALK (Cell Signaling, Danvers, MA, USA).

### EML4-ALK cloning and generation of cell lines

The native EML4-ALK variant 1 gene (Genbank: BAF73611.1; BlueSky Biotech, Worcester, MA, USA) was cloned into the retroviral vector pMSCV-Neo (Clontech, Mountain View, CA, USA), which was introduced into Ba/F3 cells by retroviral transduction. After the selection, cells were grown in the absence of IL-3. Plasmids encoding specific EML4-ALK mutations were generated with the QuikChange site-directed mutagenesis kit (Stratagene, Santa Clara, CA, USA).

### *In vitro* mutagenesis screen

Ba/F3 cells expressing native EML4-ALK were treated overnight with 100 μg/mL *N*-ethyl-*N*-nitrosourea (ENU; Sigma Aldrich, St. Louis, MO, USA) and then distributed into 96-well plates containing 250, 500, 720, 1000, 1440, or 2000 nm crizotinib. The cells were grown in standard growth medium, without IL-3, for 5 weeks. Cells from the wells containing substantial outgrowth were expanded under the original selection conditions, genomic DNA extracted, and the ALK kinase region sequenced by Taq DyeDeoxy Terminator Cycle Sequencing (Applied Biosystems, Carlsbad, CA, USA).

### Model of crizotinib bound to ALK

A homology model of ALK was built based on the crystal structure of activated insulin kinase (PDB code: 1ir3) using Prime (v2.0; Schrodinger). Crizotinib was docked into ALK using Glide SP (v5.0; Schrodinger) with postdocking minimization and the top-scoring pose chosen for further analysis.

### Subcutaneous tumor models

H3122 or Ba/F3 cells expressing EML4-ALK were implanted into the right flank of female Severe Combined Immunodeficiency (SCID) Beige mice. Crizotinib or vehicle (water) was administered once daily by oral gavage and mean tumor volume (*L* × *W *^2^ × 0.5) calculated for each group. Tumor growth inhibition (TGI) or regression (TR) was calculated as follows: TGI = (1 − Δ*T*/Δ*C*) × 100 was used when Δ*T* > 0, where Δ*T* and Δ*C* represent the mean tumor volume changes in treatment and control groups, respectively. When Δ*T* < 0, the formula TR = (Δ*T*/*T*i) × 100 was used where *T*i is the mean tumor volume for the group at the start of treatment. Tumor measurement data were analyzed with a one-way anova test (GraphPad Prism, La Jolla, CA, USA). Statistical significance was determined using Dunnett's test. P-ALK levels were measured in homogenized tumors by ELISA. Crizotinib concentrations in plasma were determined by LC/MS/MS.

## Results

**Figure 1:**
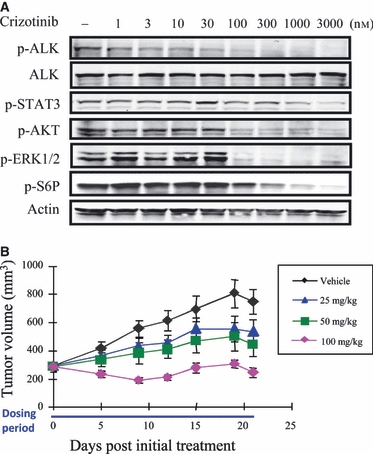
Crizotinib inhibits signaling and proliferation in EML4-ALK-dependent NSCLC cell lines. (A) immunoblot analysis of p-ALK and downstream signaling in H3122 cells treated with crizotinib. (B) *In vivo* efficacy of crizotinib in an H3122 subcutaneous xenograft model. Mean tumor volume ±SEM is plotted.

To understand the potential impact of resistant mutations on crizotinib efficacy, we first characterized its activity in *in vitro* and *in vivo* models of NSCLC. In H3122 cells, which express EML4-ALK variant 1, crizotinib inhibited ALK phosphorylation (p-ALK) with an IC_50_ of 43 nm and cell growth with a GI50 of 62 nm (Figure 1A and Table 1). This was accompanied by inhibition of p-ERK and p-S6P, although with minimal effects on STAT3 phosphorylation. Similar results were obtained with H2228 cells, which express EML4-ALK variant 3 (12). By contrast, IC_50_ values for two ALK-negative NSCLC cell lines were >1000 nm (Table 1). These data establish that crizotinib differentially inhibits the growth of EML4-ALK NSCLC cell lines relative to ALK-negative cells with approximately 10- to 20-fold selectivity.

**Table 1:** Inhibitory activity of crizotinib and TAE684 on ALK-positive and ALK-negative NSCLC lines

		Crizotinib		TAE684	
			
Cell line	ALK status	Cell growth (GI50, nm)	p-ALK (IC_50_, nm)	Cell growth (GI50, nm)	p-ALK (IC_50_, nm)
H3122	EML4-ALK v1	62 ± 18	43 ± 37	1.5 ± 0.6	1.0
H2228	EML4-ALK v3a/3b	121 ± 61	55 ± 4	3.8 ± 2	3.7
H838	ALK negative	1307 ± 270	ND	406 ± 217	ND
H23	ALK negative	1773 ± 743	ND	618 ± 283	ND

ND, not determined.

We also characterized the activity of crizotinib in a mouse H3122 xenograft model. Once daily oral administration of 25, 50, or 100 mg/kg of crizotinib for 21 days reduced tumor growth in a dose-dependent manner, with 14% tumor regression observed as the best response to treatment (Figure 1B).

To identify kinase domain mutants resistant to crizotinib, we first created a Ba/F3 cell line expressing native (unmutated) EML4-ALK variant 1. This cell line was inhibited by crizotinib with an IC_50_ of 132 nm, representing a selectivity differential of ninefold over parental Ba/F3 cells (Table 2). These assays guided us to use a crizotinib concentration range in our mutagenesis screens of 250–2000 nm. Ba/F3 cells expressing native EML4-ALK were exposed to the DNA-modifying agent ENU, cultured in 96-well plates in the presence of crizotinib dilutions and monitored for cell growth. Growth was observed in all wells containing 250 nm crizotinib. Approximately, 60% of wells at 500 nm crizotinib showed outgrowth. At higher concentrations (720, 1000, and 1440 nm), cell growth was observed in progressively fewer wells, with the only concentration showing no outgrowth being 2000 nm.

**Table 2:** Inhibitory activity of crizotinib and TAE684 on Ba/F3 cells expressing EML4-ALK mutants

	Crizotinib			TAE684	
		
	Viability (IC_50_s, nm)		p-ALK (IC_50_, nm)	Viability (IC_50_, nm)	p-ALK (IC_50_, nm)
				
Ba/F3 line	ENU clone^a^	Reintroduced	ENU clone	ENU clone	ENU clone
Parental^b^	1176 ± 282	N/A	N/A	1283 ± 348	N/A
Native EML4-ALK	132 ± 45	N/A	102 ± 70	8 ± 1	5 ± 3.5
T1151K	231 ± 106	152 ± 48	ND	ND	ND
L1152V	237 ± 25	162 ± 89	ND	ND	ND
C1156Y	489 ± 48	ND	408 ± 67	37 ± 5	11 ± 6
I1171T	393 ± 134	413 ± 62	ND	ND	ND
F1174C	479 ± 40	319 ± 90	165 ± 112	40 ± 2	6.7 ± 2.5
L1196M	981 ± 113	1215 ± 708	1162 ± 209	20 ± 3	3.3 ± 0.5
S1206R	681 ± 176	728 ± 362	356 ± 173	80 ± 9	16 ± 11
E1210K	318 ± 143	297 ± 92	ND	ND	ND
F1245C	425 ± 100	269 ± 194	ND	ND	ND
G1269S	953 ± 213	1196 ± 649	1366 ± 52	33 ± 9	35 ± 1

a ENU clone: Ba/F3 cells obtained from the crizotinib mutagenesis screen.

b Grown in the presence of 10 ng/mL IL-3.

ND, not determined; N/A, not applicable; ALK, anaplastic lymphoma kinase.

**Figure 2:**
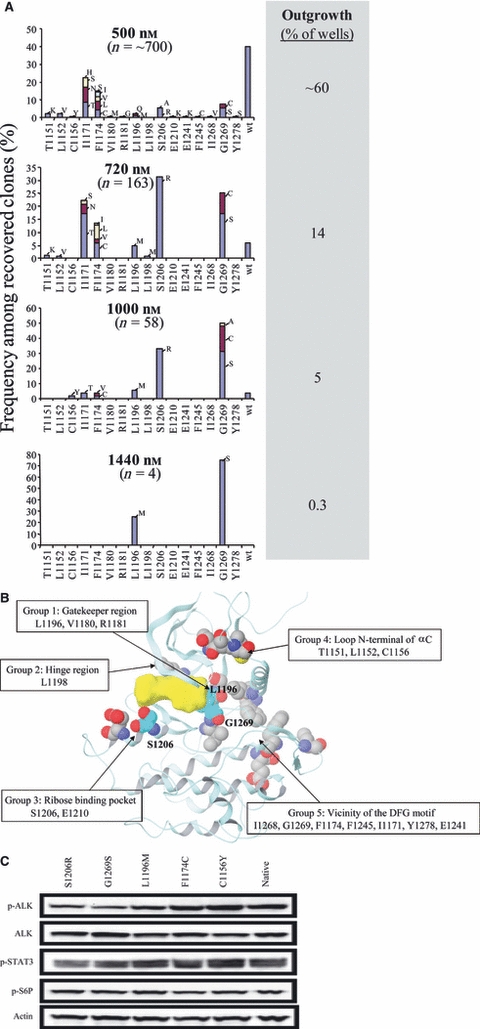
Spectrum of EML4-ALK mutations recovered in a mutagenesis screen. (A) resistant clones recovered from ENU-treated Ba/F3 cells expressing native EML4-ALK cultured with indicated concentrations of crizotinib. Each bar represents the relative percentage of the indicated EML4-ALK kinase domain mutant among recovered clones. The percentage of wells surveyed that contained outgrowth is indicated. (B) position of mutations in a model of crizotinib (shown in yellow) bound to the ALK kinase domain. (C) immunoblot analysis of p-ALK and downstream signaling in Ba/F3 cells carrying native and mutant variants of EML4-ALK.

Sequencing identified a total of 422 mutations representing amino acid exchanges at 16 different sites (Figure 2A). The spectrum of mutations was narrowed with increasing crizotinib concentrations, in terms of both the sites modified and the number of alternative amino acids identified at each position. Mutations at 15 different sites were detected at 500 nm crizotinib, eight sites at 720 nm, six sites at 1000 nm, and two sites at 1440 nm. The mutated residues identified at the highest crizotinib concentrations in our screen were C1156, I1171, F1174, L1196, S1206, and G1269. Similar results were obtained in two additional experiments.

Interestingly, one of the residues most frequently mutated in our screen, F1174, is also one of the most frequently identified positions for activating mutations in neuroblastoma (13). The same is true for the residue R1275 of ALK; yet, mutations at this site were not recovered in our screen. Consistent with this, we found that the introduction of R1275Q into EML4-ALK had no negative impact on sensitivity to crizotinib (IC_50_ 47 ± 8 nm).

We selected the ten most frequently identified mutants, each at a different residue, for further analysis (Table 2). As expected, the IC_50_ values for viability of Ba/F3 cells expressing these mutants were all above that for Ba/F3 cells expressing native EML4-ALK, with IC_50_s ranging from 231 to 981 nm. The three most resistant mutants, L1196M, S1206R, and G1269S, all had IC_50_s within twofold of parental, ALK-negative, Ba/F3 cells (IC_50_ 1176 nm). There was no evidence that the mutations increased the basal activity of ALK as assessed by levels of p-ALK and downstream signaling proteins (Figure 2C). However, the ability of crizotinib to inhibit ALK phosphorylation was substantially reduced in all mutants tested, with IC_50_s >1000 nm for L1196M and G1269S (Table 2). A similar impairment was seen on the ability of crizotinib to inhibit downstream signaling in cells expressing the L1196M mutant (Figure S2A). We verified that crizotinib resistance was solely dependent on the mutations by analyzing Ba/F3 cell lines expressing mutant EML4-ALK fusions generated by site-directed mutagenesis and observing similar results (Table 2, ‘Reintroduced’).

The 16 mutations are located around the kinase active site and can be categorized into five groups, involved in either direct or indirect contacts with crizotinib. As shown in Figure 2B, group 1 is located around the gatekeeper position, group 2 is located around the kinase hinge region, group 3 is located near the ribose binding pocket, group 4 is located at the loop of the N-terminus of α-helix C, and group 5 is located in the vicinity of the kinase DFG (Asp-Phe-Gly) motif of the activation loop. The three mutations that conferred the strongest resistance were the L1196M gatekeeper residue, S1206R at the solvent front, and G1269S near the DFG motif.

**Figure 3:**
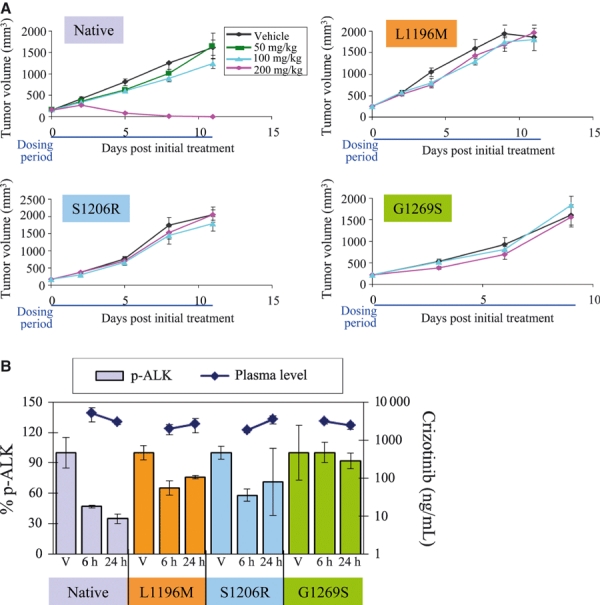
Crizotinib is not efficacious in mouse Ba/F3 xenograft models expressing EML4-ALK mutants. (A) *In vivo* efficacy of crizotinib in EML4-ALK-driven Ba/F3 models. Mean tumor volume ±SEM was plotted. (B) PK/PD analysis after treating tumor-bearing mice with a single dose of vehicle (V, for 6 h) or 200 mg/kg crizotinib.

We characterized the sensitivity of these three mutants in mouse xenograft studies (Figure 3A). Ba/F3 cells expressing native EML4-ALK grew robustly as subcutaneous xenografts in SCID mice. Daily oral treatment of these mice with crizotinib at 100 mg/kg induced a modest tumor growth inhibition of 33%, which was not statistically significant (p > 0.05), and 200 mg/kg [the highest reported daily oral dose for crizotinib in mice (14)] induced complete regressions by 12 days of treatment. However, analogous Ba/F3 xenografts expressing L1196M, S1206R, or G1269S mutants were completely insensitive to these doses, with no statistically significant changes in tumor growth rate.

In pharmacodynamic studies (Figure 3B), xenografts expressing native EML4-ALK exhibited a 60–70% inhibition in p-ALK levels at 6 h postdose, with more pronounced inhibition at 24 h. By contrast, p-ALK levels were reduced by approximately 25–35% at 6 h in tumors expressing L1196M or S1206R, with a partial recovery at 24 h. There was no significant inhibition in tumors expressing the G1269S mutation. Drug exposure was similar in all models, confirming that crizotinib inactivity in the mutant ALK efficacy studies is because of the inadequate target inhibition.

TAE684 is a previously described ALK inhibitor (11) that we have confirmed to be substantially more potent and selective than crizotinib in ALK-driven NSCLC models (Table 1). TAE684 inhibited the viability of Ba/F3 cells expressing native EML4-ALK (IC_50_ 8 nm) or the five mutants that conferred the greatest resistance to crizotinib (IC_50_s of 20–80 nm) all with substantial selectivity over parental, ALK-negative Ba/F3 cells (IC_50_ 1283 nm; Table 2). Potent inhibition of p-ALK and downstream signaling was also observed (Table 2; Figure S2).

## Discussion

In this study, we have used an accelerated mutagenesis strategy to identify an extensive set of mutations in ALK that can confer resistance to crizotinib. Alterations at 16 different amino acids were observed, with three of them, L1196M, S1206R and G1269S, rendering cells completely insensitive in mouse xenograft studies. Interestingly, use of an alternative approach, in which an ALK-positive NSCLC cell line is exposed to increasing doses of crizotinib, led to the identification of one mutation, L1196M, that could confer resistance to crizotinib (15). Our results confirm that kinase domain mutations are a potential mechanism for acquired resistance to crizotinib and identify a novel, sizable panel of specific candidate mutations for correlation with clinical studies.

An important factor in the resistance susceptibility of crizotinib appears to be its relatively narrow window of activity against ALK-positive versus ALK-negative cell lines: a differential of approximately 10- to 20-fold in our studies (Tables 1 and 2). This means that even modest potency reductions linked to single mutations may abrogate the selective activity of the compound. Ultimately, the range of ALK mutations observed clinically will depend on pharmacologic considerations, such as drug exposure and target inhibition levels in patients. By analogy with CML (16), however, more potent ALK inhibitors should be able to overcome crizotinib-resistant mutants. Indeed, we show that a more potent and selective ALK inhibitor, TAE684, maintains substantial activity against the mutations that confer the greatest resistance to crizotinib, with all mutants inhibited with at least 15-fold selectivity over ALK-negative cells. Recently, three additional ALK inhibitors, AP26113 (15), CH5424802 (17), and X-396 (18), have also be shown to be capable of inhibiting the L1196M variant of ALK in preclinical studies. Consistent with our observations regarding TAE684, each of these compounds has also been shown to be a more potent and selective inhibitor of ALK than crizotinib.

Most of the mutations can be rationalized based on structural analysis (Figure 2B). The L1196M gatekeeper mutation likely sterically impedes crizotinib binding. S1206, located near the ribose binding pocket of ATP, makes a contact with crizotinib, in the docked model, that would be eliminated by the S1206R mutation. Finally, G1269 (with D1268) forms a small hydrophobic pocket that binds the 3-fluoro-2,6-dichlorophenyl group of crizotinib. This interaction would be disrupted by the G1269S mutation. Other mutated residues likely stabilize the conformation of the crizotinib contact residues, including V1180 and R1181 (in contact with L1196); E1210 (in contact with S1206); and D1268, F1174, F1245, I1171, Y1278, and E1241 (in direct or indirect contact with G1269). The three residues in group 4 (T1151, L1152, and C1156) do not make direct contacts with crizotinib, but likely have indirect conformational roles. TAE684, on the other hand, has limited molecular contact interactions with the gatekeeper residue L1196 as well as with G1269 of the DFG motif, according to the recently published crystal structure (19), and is thus less susceptible to these two mutations. However, TAE684 is quite sensitive to the S1206R mutation. Analysis of the crystal structure indicates that the mutated arginine 1206 is likely to form a stabilized side chain conformation by interacting with its neighboring two acidic residues (D1203 and E1210), and such a conformation may be incompatible with the optimized binding pose of TAE684 in the ALK protein.

Several isolated mutations were at positions where activating mutations have previously been identified in ALK-expressing neuroblastoma (F1174, T1151, I1171, F1245, and Y1278) (13,20,21). In particular, F1174 is one of the most frequently mutated residues in neuroblastoma, and the mutations of F1174 to Cys, Val, Ile, and Leu were observed in our screen. F1174 is at the loop C-terminal to the alpha-helix C and forms a hydrophobic patch with neighboring residues including F1241 of the DFG motif. F1174L may therefore stabilize an active conformation that is both more oncogenic and less favored for crizotinib binding.

This screen has several potential limitations. Ba/F3 cells *in vitro* are unlikely to faithfully recapitulate the cellular context of ALK-driven primary human tumors. Additionally, mutation-focused screens do not probe alternative resistance mechanisms, such as gene amplification or upregulation of parallel signaling pathways. Nevertheless, such screens have proved highly predictive with other kinases (10). Most importantly, the clinical relevance of our findings is supported by the recent identification, after completion of our study, of the L1196M and C1156Y mutations from a patient with NSCLC with acquired resistance to crizotinib (22) and a separate report identifying the F1174L mutation in an IMT patient with similar acquired resistance (23). Our studies suggest multiple additional mutations that have the potential to confer resistance to crizotinib in patients and provide guidance for the rational design and optimization of potent and selective second generation drugs that may be able to overcome ALK-based mechanisms of resistance.

## Supporting Information

**Figure S1.** Chemical structure of crizotinib and TAE684.

**Figure S2.** Impact of L1196M on anti-ALK activity of crizotinib and TAE684.
