# Supplementary figures and images for: Crizotinib-Resistant Mutants of EML4-ALK Identified Through an Accelerated Mutagenesis Screen
Source: Chem Biol Drug Des. 2011 Dec;78(6):999–1005. doi: 10.1111/j.1747-0285.2011.01239.x (PMC3265718; doi:10.1111/j.1747-0285.2011.01239.x)

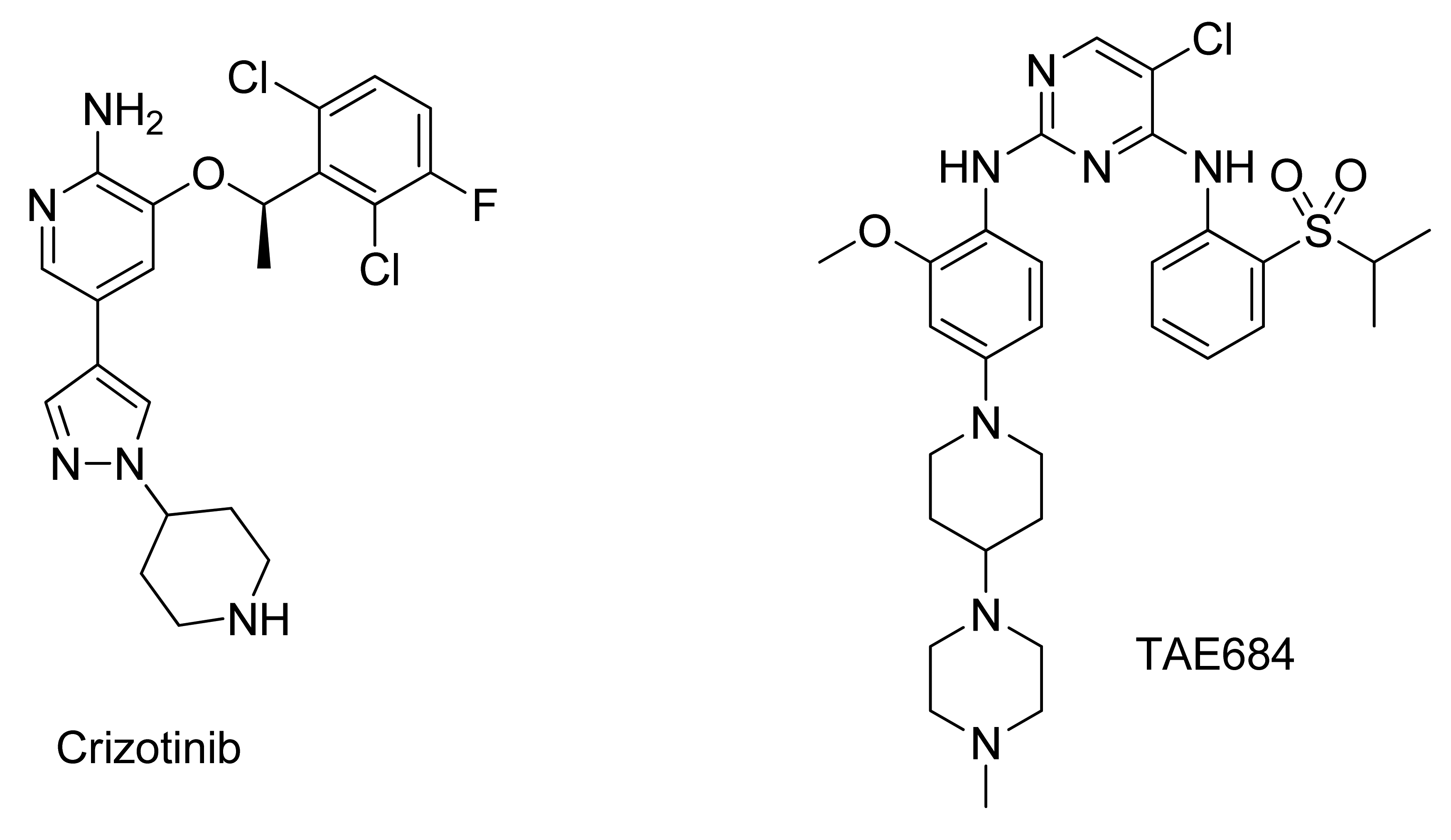

Supplement: Supplementary file 1 [file cbdd0078-0999-SD1.tif]

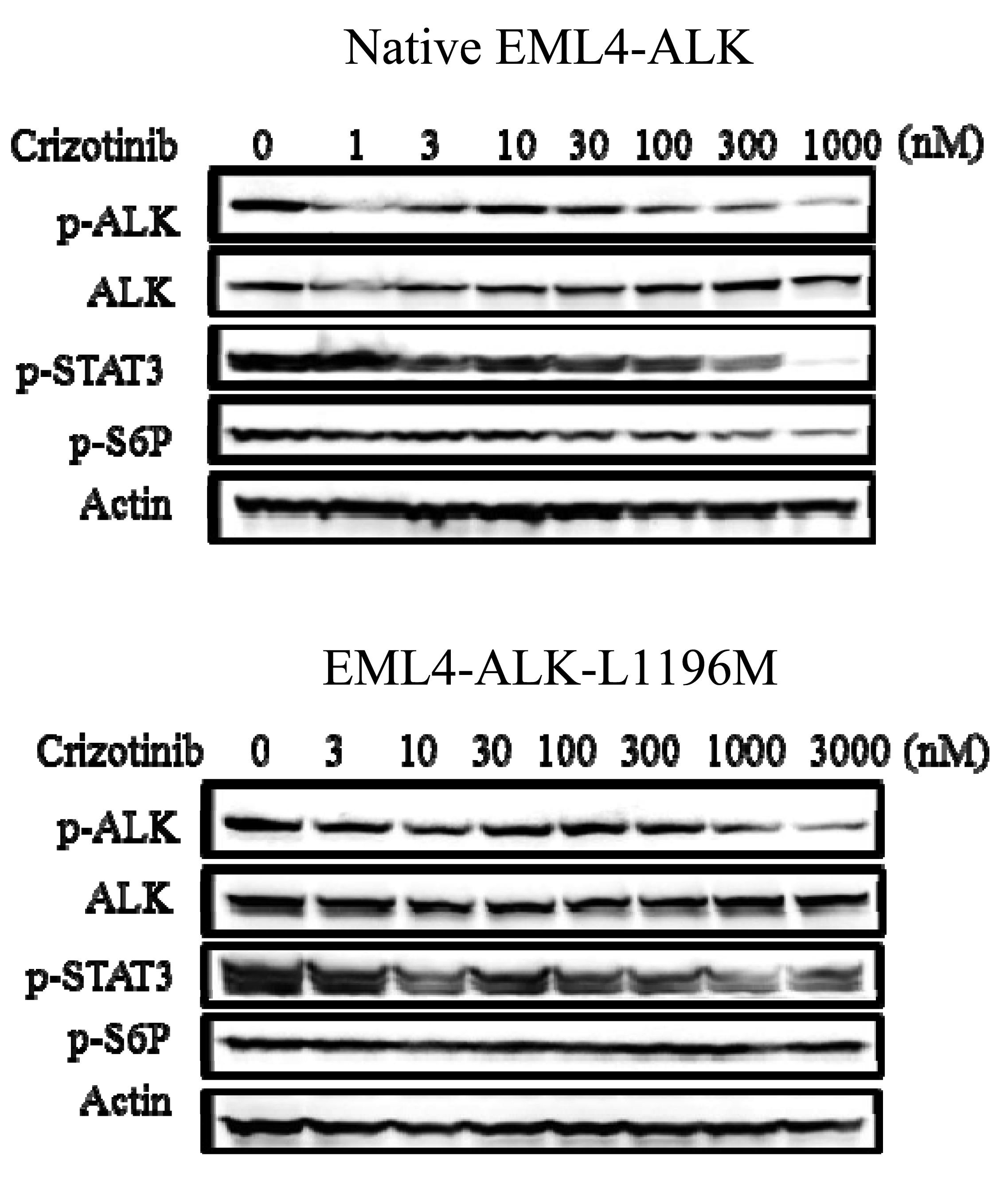

Supplement: Supplementary file 2 [file cbdd0078-0999-SD2.tif]

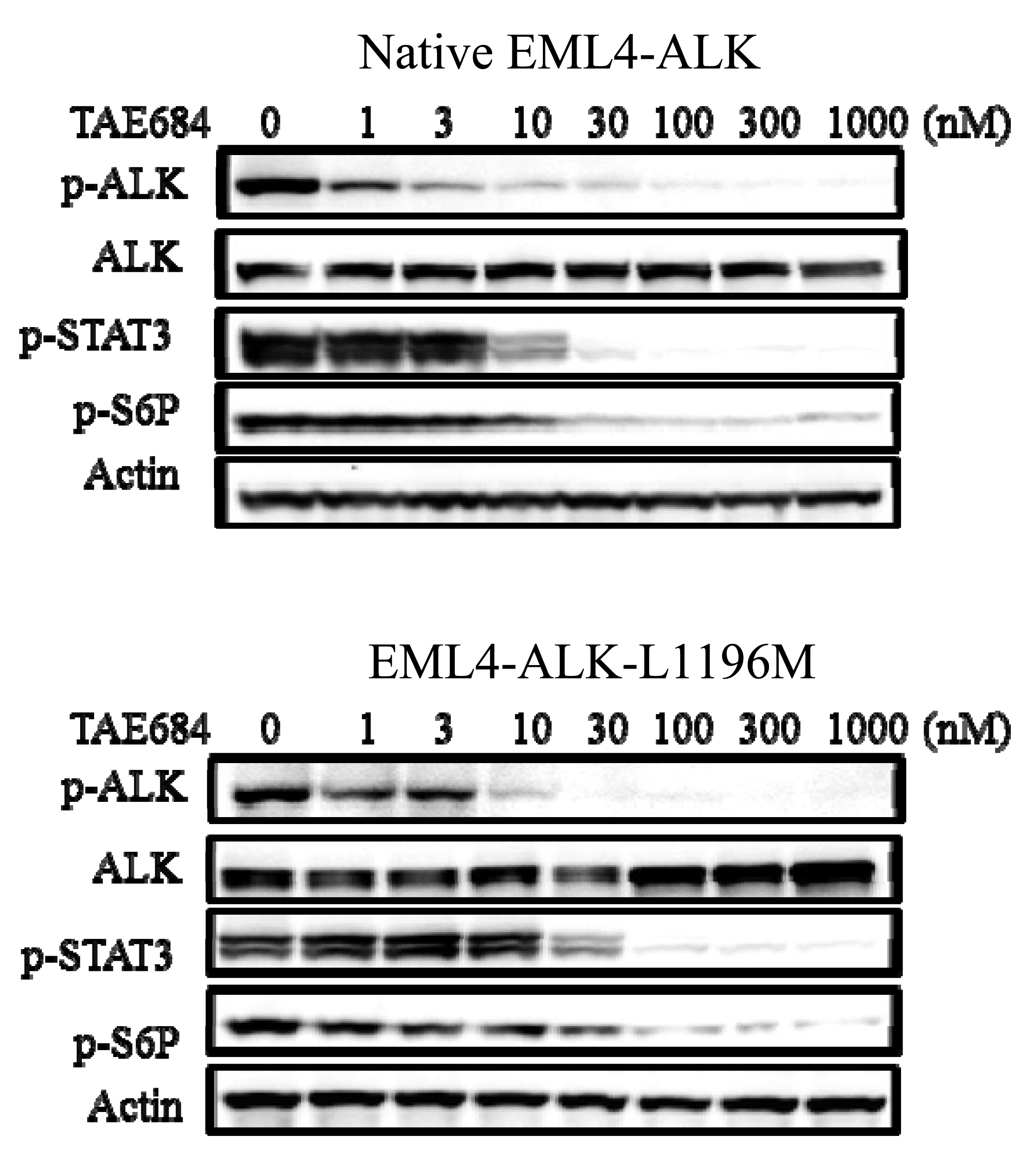

Supplement: Supplementary file 3 [file cbdd0078-0999-SD3.tif]
