# Supplementary material for: Crizotinib-Resistant Mutants of EML4-ALK Identified Through an Accelerated Mutagenesis Screen
Source: Chem Biol Drug Des. 2011 Dec;78(6):999–1005. doi: 10.1111/j.1747-0285.2011.01239.x (PMC3265718; doi:10.1111/j.1747-0285.2011.01239.x)
Supplement: Supplementary file 4 [file cbdd0078-0999-SD4.doc]

**Supplementary figure legends**

**Supplementary Figure 1.** Chemical structure of crizotinib and TAE684.

**Supplementary Figure 2.** Impact of L1196M on anti-ALK activity of crizotinib and TAE684. Immunoblot analysis of p-ALK and downstream signaling in Ba/F3 cells carrying native or L1196M-EML4-ALK after treatment with crizotinib (A) or TAE684 (B).
